# Application of miniprobe endoscopic ultrasonography in endoscopic submucosal dissection surgery for laterally spreading tumor of the rectum: a case report

**DOI:** 10.3389/fonc.2025.1673744

**Published:** 2025-12-03

**Authors:** Lihong Li, Qianbao Lv, Tianpeng Zhang, Jinsong Lai, Dajian Zhu

**Affiliations:** 1 Department of Gastrointestinal Surgery, Shunde Women and Children’s Hospital of Guangdong Medical University (Maternal & Child Healthcare Hospital of Shunde Foshan), Foshan, China; 2 Department of Gynaecology, Shunde Women and Children’s Hospital of Guangdong Medical University (Maternal & Child Healthcare Hospital of Shunde Foshan), Foshan, China

**Keywords:** miniprobe endoscopic ultrasonography, laterally spreading tumor, rectum, endoscopic submucosal dissection, case report

## Abstract

**Background:**

Laterally spreading tumor (LST) is a special clinical lesion occurring in the colorectum. Generally before surgery, it is necessary to identify whether the rectal submucosa and the muscularis propria are invaded or not. Miniprobe endoscopic ultrasonography (MEUS) is a convenient and advanced method for diagnosis of LST invasion of the wall of the rectum. Herein, we present a case of LST in the rectum to demonstrate the application of MEUS.

**Case description:**

A 49-year-old male patient who was found to have a flat mass in the rectum during an enteroscopy for physical examination, the pathological report of the biopsy of the LST revealed high-grade intraepithelial neoplasia, so the patient was admitted to our hospital. A systematic computed tomography(CT) scan showed slight thickening of the local rectal wall, with no evidence of regional lymph node metastasis or distant metastasis. To determine the depth of tumor invasion, MEUS was performed first, the result showed that the tumor, approximately 4 cm in diameter, was confined to the mucosal layer of the rectum, with intact submucosa and intrinsic muscular layer from the MEUS. Based on these findings, endoscopic submucosal dissection (ESD) was successfully performed. Final pathological diagnosis confirmed high-grade intraepithelial neoplasia with focal carcinomatosis, but the focal carcinoma did not invade the muscular layer of the rectal mucosa, both the horizontal and basal margins of the LST were negative for malignancy.

**Conclusion:**

MEUS is an important and useful diagnostic method for identifying the depth of the invasion of LST in the rectum before ESD surgery, and ESD is an effective and safe procedure in the treatment of colorectal LSTs.

## Introduction

Laterally spreading tumor (LST) is a special clinical lesion, and endoscopic submucosal dissection (ESD) is an effective treatment method (1, 2). To achieve complete excision and avoid tumor residue, it is important to determine the depth of the tumor invasion. Miniprobe endoscopic ultrasonography (MEUS) is a convenient and effective diagnostic method before surgery for LST. To our best knowledge, there is no case report about the application of MEUS in LST of rectum.

## Case description

**Figure 1:**
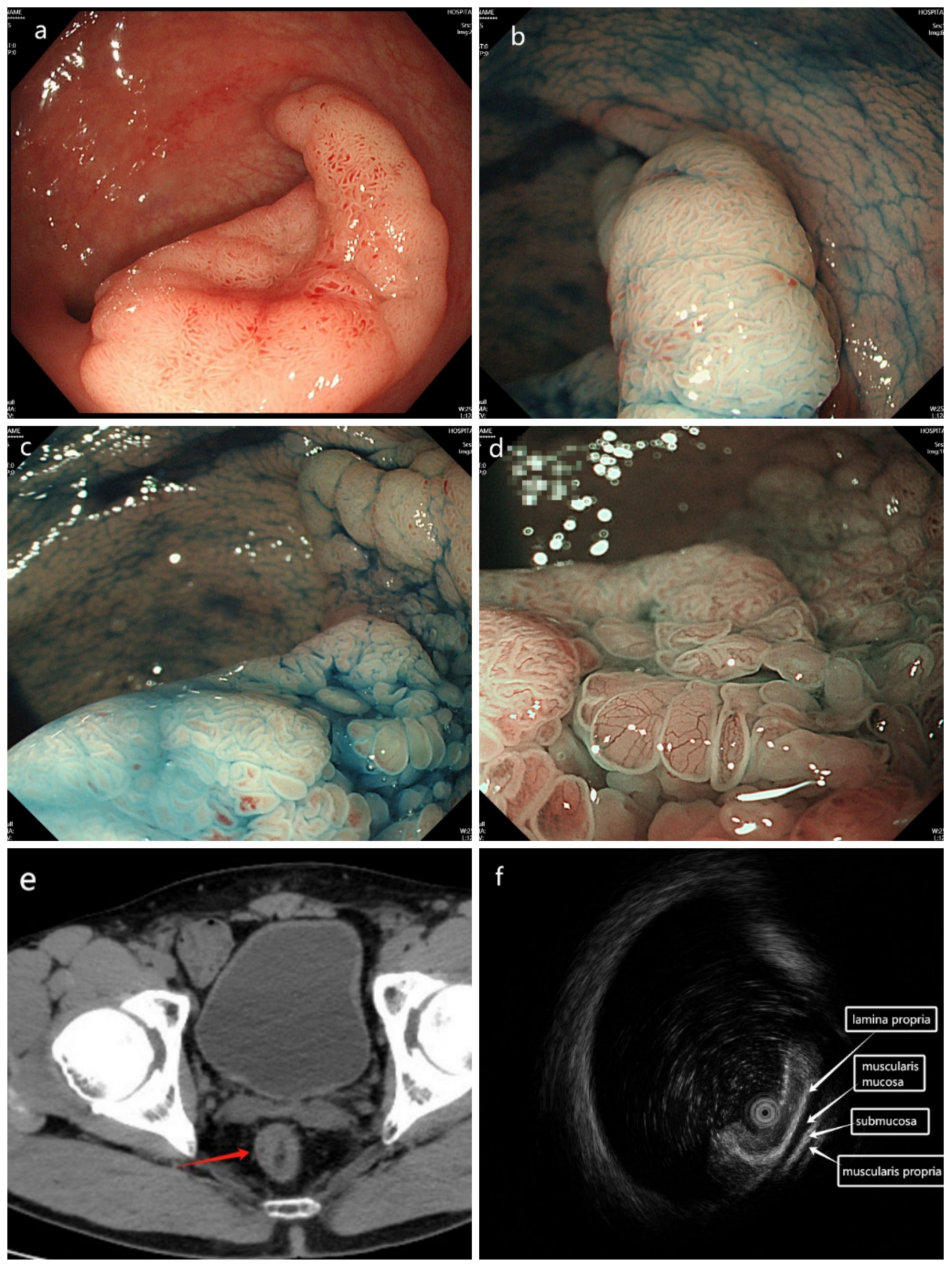
Colonoscopy of the tumor’s images. **(a)** The colonoscopy showed a flat tumor about 4 cm in diameter with central slight depression located at the right posterior wall of the proximal rectum about 7 cm from the anal verge. **(b, c)** The staining image of indigo showed that the classification of pit pattern was IIIL-IV. **(d)** Magnifying endoscopy narrow band imaging(ME-NBI) reveals that the microvessel (MV) was slightly dilating, the microsurface remained regular. **(e)** Computed tomography (CT) image showed that the local mucosa of the right posterior wall of the rectum was thickened slightly. **(f)** The images of miniprobe endoscopic ultrasonography revealed the laterally spreading tumor was confined to mucosal layer of the rectum, the submucosa and intrinsic muscular layer was intact.

**Figure 2:**
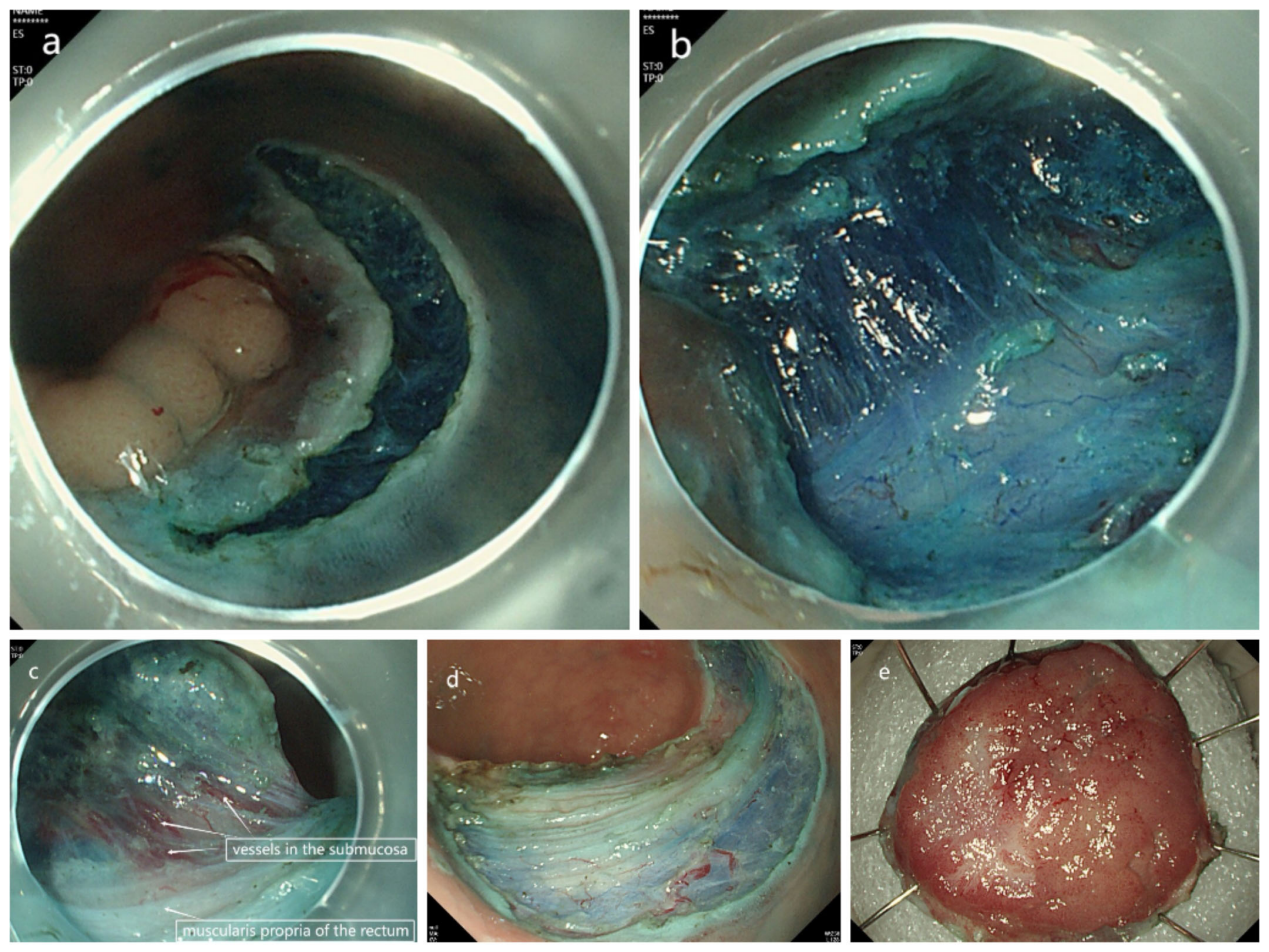
The images of the endoscopic submucosal dissection surgery procedure. **(a)** Dissecting the rectal mucosa along 3 mm away from the verge of the LST. **(b)** The image of the submucosa of the LST during the procedure of ESD surgery. **(c)** the image of the vessels in the submucosa and the muscularis propria of the rectum. **(d)** the image of the postoperative wound of the rectum, the muscularis propria of the rectum was shown clearly. **(e)** the image of the laterally spreading tumor after ESD surgery.

**Figure 3:**
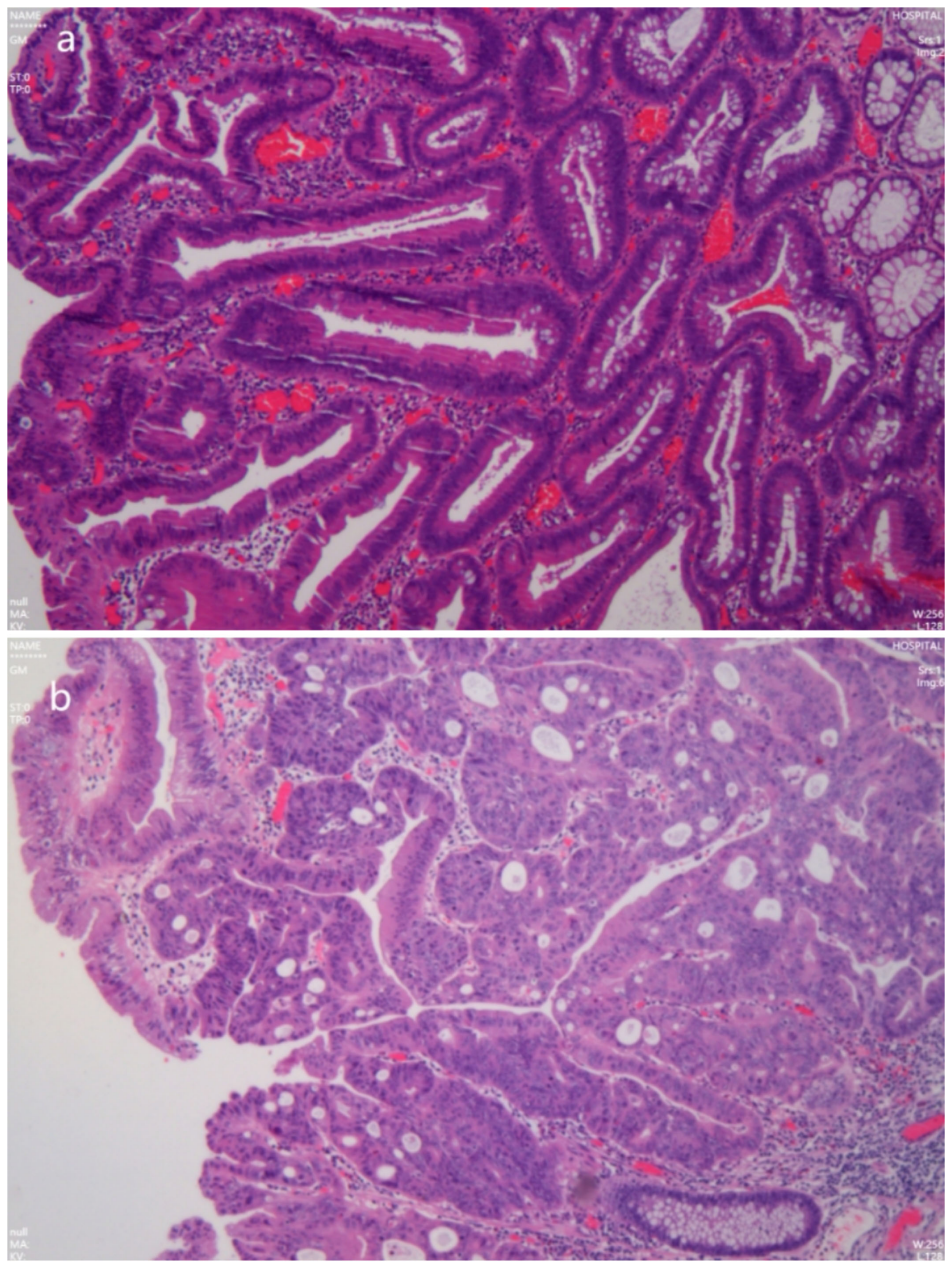
Hematoxylin and eosin staining for laterally spreading tumor revealed that tumor was tubular villous adenoma with high-grade intraepithelial neoplasia with local carcinomatosis, and local cancer limited to the muscularis mucosa [**(a)** original magnification×100, **(b)** original magnification×400].

A 49-year-old male patient had a flat mass in the rectum revealed during an enteroscopy for physical examination. the biopsy pathological examination showed high-grade intraepithelial neoplasia (HGIN). The tumor was diagnosed as LST located in the rectum about 7 cm from the anal verge, and the classification was LST-NG(LST non-granular type) (**Figure 1A**). So, the patient was admitted to our hospital for further examination and treatment. He had been previously in good health and had no medical history. After admission, further enteroscopy examination was performed. The tumor was approximately 40 mm in diameter, and the staining image of indigo (specification number is MTN-DYZ-15, lot number is MMAA230115252) showed that the classification of pit pattern was IIIL-IV (**Figures 1B, C**). Magnifying endoscopy narrow band imaging (ME-NBI) revealed that the microvascular (MV) was slightly dilated and the microsurface remained regular (**Figure 1D**). A systematic computed tomography (CT) scan showed that the local mucosa of the rectum was slightly thickened (**Figure 1E**). There was no distant metastasis to any other organs and no evidence of regional lymph node metastasis. To determine the depth of the tumor invasion, we needed to perform endoscopic ultrasonography (EUS) before surgery. We used a new brand endoscopic ultrasound miniprobe (miniprobe model: MD-886, 20MHz, serial number: 152DB12C, ultrasound equipment model: IMP-8901, serial number: 152D734A, INNERMED, Guangdong, China.). The MEUS images showed that the tumor was confined to the mucosal layer of the rectum, and the muscularis mucosa, the submucosa and muscularis propria of the rectum were intact (**Figure 1F**). Therefore, ESD surgery was successfully performed on the patient (**Figures 2A-E**). The final pathological diagnosis of the LST specimen was high-grade intraepithelial neoplasia with local carcinomatosis. However, the local carcinoma was confined to the rectal mucosa and did not break through the muscularis mucosa, and the horizontal and basal margin of the LST specimen were normal (**Figures 3A, B**).

## Discussion

Laterally spreading tumor (LST) is a kind of special lesion occurring on the colorectal wall. It is regarded as an aggressive type of colorectal neoplasm, which is more than 10 mm in diameter in most cases. It grows laterally instead of vertically along the colorectal wall and has the tendency of malignant change. Most patients with LST have no symptoms and are discovered by chance during a physical examination. The proportion of LST among colorectal neoplasms was 1.52% and 1.38% in asymptomatic and average-risk Chinese groups (3).

According to the morphology under the endoscopy, the LSTs are classified into two types: granular type (LST-G) and non-granular type (LST-NG). The former type is divided into the homogeneous G-type and nodular mixed G-type, while the latter includes the flat elevated NG-type and a pseudo-depressed NG-type, these four subtypes show distinct clinicopathological characteristics (4). The distribution of high-grade intraepithelial neoplasia (HIGN) and submucosal carcinoma is more prominent among granular nodular mixed tumors compared to the other three subtypes (4, 5). Some studies have revealed that the incidence of LSTs progressing to HIGN was from 20.9% to 33.8% (6, 7). Although LST is less likely to grow vertically, it can also develop into submucosal invasive cancer (2). Most probably, the subtype of pseudo-depressed lesions shows the highest incidence of submucosal carcinoma, and there are no difference in pathology between the lesions of homogeneous G-type and flat elevated NG-type (4).

In this case, the tumor measured approximately 40 mm in diameter and was confined to the mucosal layer without invading the submucosa on the MEUS images. Its classification was LST-G (H) according to the morphology under the white light endoscopy. Microvascular expansion was observed and the microsurface remained regular by ME-NBI, and the staining image of indigo showed the classification of pit pattern was IIIL-IV. These findings indicated that the tumor was most likely a HGIN and local cancer could not be ruled out. Based on the above results, the ESD surgery was the first choice rather than surgical operation.

But before surgery, it was necessary to further understand the invasion depth of the tumor or whether the tumor had invaded the rectal submucosa. Many researchers have pointed out that MEUS played an important role in assessing T and N stages of colon cancer. Specifically, when assessing the presence or absence of lymph node metastasis, miniprobes achieve an accuracy of 82% and a sensitivity of 67%. MEUS is a valuable addition to routine endoscopy and can provide crucial treatment guidance for suspected early gastric cancer (EGC) and colorectal lesion (8, 9).

In this case, the MEUS was performed on the patient. We found that the tumor was confined to the rectal mucosa and did not break through the muscularis mucosa. The submucosa and muscularis propria of the rectum were intact from the MEUS images, and there was no lymph node metastasis. Therefore, it was feasible to perform the ESD surgery according to the MEUS, the LST was completely removed along with a 3 mm normal mucosal margin around it, the final pathological diagnosis of the LST specimen was HGIN with local carcinomatosis, but the local carcinoma did not invade the muscularis mucosa of the rectum, the horizontal and basal margin of the LST specimen were normal.

A report discovered that the expression levels of lipocalin-2 (LCN-2) and matrix metallopeptidase-9 (MMP-9) were significantly higher in LSTs, and it concluded that LCN-2 and MMP-9 were associated with the disease progression in LSTs, including lateral growth and vertical invasion. Furthermore, the report also found that the LCN-2 and MMP-9 were correlated with worse pathological grading. The researchers in the report suggested that LCN-2 was a potential biomarker for LST disease progression and might be a novel therapeutic target in LSTs (10).

## Conclusion

The LST is a special lesion occurring on the colorectal wall, which has the potential to become malignant and needs to be treated by surgery. The MEUS is an important and effective technique to assess the invasion depth of LST, which could provide treatment guidance in choosing the surgical method (ESD surgery or radical surgery for tumor). The expression levels of LCN-2 and MMP-9 were associated with the disease progression in LSTs.

## Data Availability

The original contributions presented in the study are included in the article/Supplementary Material. Further inquiries can be directed to the corresponding author.
